# Core Imaging Library - Part II: multichannel reconstruction for dynamic and spectral tomography

**DOI:** 10.1098/rsta.2020.0193

**Published:** 2021-08-23

**Authors:** Evangelos Papoutsellis, Evelina Ametova, Claire Delplancke, Gemma Fardell, Jakob S. Jørgensen, Edoardo Pasca, Martin Turner, Ryan Warr, William R. B. Lionheart, Philip J. Withers

**Affiliations:** ^1^ Henry Royce Institute, Department of Materials, The University of Manchester, Manchester, UK; ^2^ Department of Mathematics, The University of Manchester, Manchester, UK; ^3^ Research IT Services, The University of Manchester, Manchester, UK; ^4^ Scientific Computing Department, Science Technology Facilities Council, UK Research and Innovation, Rutherford Appleton Laboratory, Didcot, UK; ^5^ Department of Mathematical Sciences, University of Bath, Bath, UK; ^6^ Department of Applied Mathematics and Computer Science, Technical University of Denmark, Kongens Lyngby, Denmark; ^7^ Laboratory for Applications of Synchrotron Radiation, Karlsruhe Institute of Technology, Karlsruhe, Germany

**Keywords:** X-ray CT, sparse CT, positron emission tomography (PET), magnetic resonance imaging (MRI), materials science, computed tomography, inverse problems, iterative reconstruction

## Abstract

The newly developed core imaging library (CIL) is a flexible plug and play library for tomographic imaging with a specific focus on iterative reconstruction. CIL provides building blocks for tailored regularized reconstruction algorithms and explicitly supports multichannel tomographic data. In the first part of this two-part publication, we introduced the fundamentals of CIL. This paper focuses on applications of CIL for multichannel data, e.g. dynamic and spectral. We formalize different optimization problems for colour processing, dynamic and hyperspectral tomography and demonstrate CIL’s capabilities for designing state-of-the-art reconstruction methods through case studies and code snapshots.

This article is part of the theme issue ‘Synergistic tomographic image reconstruction: part 2’.

## 1. Introduction

Over recent years in X-ray computed tomography (CT), there has been a growing interest in dynamic and spectral CT thanks to the technological advancements on detector speed and sensitivity and on multichannel photon-counting detectors (PCDs), as depicted in the EPSRC Tomography roadmap [1].

In dynamic CT, the aim is to reconstruct a series of images and depict the complete spatio-temporal response of the scanned object. These temporal variations may occur because the composition/structure evolved, e.g. corrosion, or the object is subject to external input, e.g. compression, or the object moved during the scanning process.

In spectral CT, using a pixelated energy sensitive detector, it is possible to collect *n* energy-specific radiographs, where *n* is the number of energy channels. As a result for any voxel in the system, it is possible to reconstruct the profile of attenuation coefficient as a function of energy, or conversely to create a tomogram corresponding to each energy bin. Since each chemical element has a characteristic attenuation profile this provides a fingerprint of the elements in each voxel. This fingerprint is especially clear for attenuation spectra that includes the energies corresponding to the X-ray absorption edges (K-edges) for the elements concerned, because there is an abrupt change in attenuation on either side of the edge [2]. Moreover, in pulsed neutron imaging data, sharp edges can also be imaged [3]. In this case, the edges, i.e. the Bragg edges, correspond to abrupt increases in the transmitted spectrum, when the energy is below that possible for Bragg diffraction out of the beam for each diffraction peak providing unique fingerprints corresponding to different crystal structures. In general, for spectral imaging, because the signal is allocated to a number of energy bins rather than accumulated to give a single image, the energy-resolved data acquired usually suffer from low signal-to-noise ratio, acquisition artefacts and angular undersampling making tomographic image reconstruction difficult.

The scope of this paper is to present the capabilities of the Core Imaging Library (CIL) (https://www.ccpi.ac.uk/cil), releases available at [4,5], of the collaborative computational project in tomographic imaging (CCPi) for multichannel tomography. It allows one to reconstruct higher quality images and ensure more accurate spatiospectral K-edge identification, see for instance [6,7], where novel reconstruction methods are introduced for laboratory-based hyperspectral CT and neutron tomography, respectively. CIL is an open source, object-oriented library (primarily written in Python) for processing tomographic data. We can read, load, preprocess, reconstruct, visualize and analyse tomographic data from different applications, e.g. X-ray CT, X-ray laminography, neutron tomography (NT) and positron emission tomography (PET).

### (a) Outline of the paper

In the first section, we give a brief overview of CIL and introduce notation for the optimization framework necessary for the reader to make the transition from mathematical formulation to code. Then, we consider a simple exemplar case study involving two simple three-channel imaging tasks, i.e. colour denoizing and inpainting. For the first task, the aim is to solve the total variation (TV) denoizing problem using the fast gradient projection (FGP) algorithm [8]. For the inpainting problem, we use the total generalized variation (TGV) regularization and solve it using the primal-dual hybrid gradient algorithm (PDHG) [9]. In the following sections, we consider two real tomography applications, namely dynamic X-ray and hyperspectral CT. In §4, we focus on dynamic tomographic imaging with severely limited projection data. We compare different regularizers defined over the spatio-temporal domain and under different undersampled acquisition geometries, including Tikhonov, TV and directional total variation (dTV) regularizers. In the final example, we deal with four-dimensional hyperspectral tomographic data. We use a stochastic version of the PDHG [10], with TV regularization to reconstruct the data with different coupling between spatial and spectral dimensions.

## 2. Core imaging library

### (a) Overview

In *Core Imaging Library - Part I* [4], we described the main building blocks of CIL: **cil.framework**, **cil.optimization**, **cil.processors**, **cil.io** and **cil.utilities**. We illustrated the basic usage of CIL data structures, as applied to a number of X-ray CT cases with different geometries, e.g. parallel, cone and laminography and also different modalities such as NT and PET. CIL wraps a number of third-party libraries, using **cil.plugins**, to perform various operations required for CT reconstruction. For instance, we can use the Astra-Toolbox [11] or TIGRE [12], to perform forward and backward projection steps, filtered back projection (FBP) and Feldkamp–Davis–Kress (FDK) reconstructions for different acquisition geometries and can use the CCPi-Regularization Toolkit (CCPi-RGL) [13], to employ several regularizers with a CPU/GPU hardware acceleration. In addition, CIL is designed such that the data structures of the Synergistic Image Reconstruction Framework (SIRF) [14], from the collaborative computational project in synergistic reconstruction for Biomedical Imaging (CCP-SynerBi), www.ccpsynerbi.ac.uk, can be used for PET and magnetic resonance imaging (MRI) reconstruction [15].

### (b) Optimization framework

The **cil.optimization** framework contains three structures, namely Function, Operator and Algorithm that formalize a generic optimization problem for imaging applications as

2.1 $$u^{*}=\underset{u\in X}{\text{arg min}}f(Ku)+g(u)\equiv \underset{u\in X}{\text{arg min}}\sum_{i=0}^{n-1}f_{i}K_{i}(u)+g(u).$$

We let X, Y denote finite-dimensional vector spaces, K:X→Y a linear operator with operator norm $||K||=\max\{||Ku|{|}_{Y}:||u|{|}_{X}\leq 1\}$ and proper, convex functions $f:Y\to \bar{R}$,^1^ $g:X\to \bar{R}$. Note that in certain cases, it is convenient to decompose $Y=Y_{0}\times \ldots \times Y_{n-1}$, *n* ≥ 1 and consider a separable function $f(y):=f(y_{0},\ldots ,y_{n-1})=\sum_{i=0}^{n-1}f_{i}(y_{i})$ which results in the right-side formulation in (2.1).

In the following case studies, using different definitions for the triplet (*K*, *f*, *g*), we can express optimization problems for several imaging tasks. For example, in denoising, we let *K* be the identity operator, in inpainting it is a mask operator that encodes missing pixel information, while it is a projection operator for tomography. The functions *f*, *g* allow us to define a fidelity term, that measures the distance between the acquired data *b* and the forward-projected reconstruction image as well as a regularizer, which enforces a certain regularity on *u*. If the noise follows a Gaussian distribution an appropriate choice for the fidelity term is $||Kx-b|{|}_{2}^{2}$. In the case of impulse noise, the *L*^1^ norm $||Kx-b|{|}_{1}$ leads to more efficient restorations and for Poisson noise the Kullback–Leibler divergence ∫Kx−blog⁡Kx is the most suitable choice. The choice of the regularizer, e.g. Tikhonov, TV and TGV, favours minimizers of (2.1) with certain geometric features and is usually weighted by positive parameters to control the influence between data fidelity and regularization terms.

In this paper, we mainly focus on *model-based* variational problems, meaning that the forward operator *K* and the probability distribution of the observational noise are known *a priori*. In particular, for X-ray CT [4], PET or MRI [15] applications, noise distribution and hence the appropriate distance functions are well established, see [16]. CIL may also be employed in an ad hoc fashion if the noise type is unknown, to experiment with which norm provides the best reconstruction result empirically, as shown in [17]. More specific blind noise methods are an active research beyond the current scope of CIL and we hope to expand in these directions, within a general data-driven framework in the future, see for instance [18] and references therein.

In order to find an approximate solution for minimization problems of the (2.1) form, we use a different CIL Algorithm for smooth and non-smooth objective functions such as the conjugate gradient least squares (CGLS), simultaneous iterative reconstruction technique (SIRT) and proximal type algorithms, which are extensively used in this paper, such as the FGP and the PDHG, SPDHG algorithms. In the FGP algorithm, we require that the function *g* has a proximal operator defined as

2.2 $${\mathrm{prox}}_{\tau g}(u):=\underset{v}{\text{arg min}}\frac{1}{2}||v-u|{|}_{2}^{2}+\tau g(v),$$

which has a ‘simple’ closed form solution or can be computed efficiently numerically. Also, we assume that *f* is continuously differentiable and has Lipschitz continuous gradient *L*. On the other hand, in the PDHG algorithm, we allow functions *f* and *g* to be non-differentiable and express (2.1) into a *saddle point problem*,

2.3 $$\min_{u\in X}\max_{z\in Y}\left\langle Ku,z\right\rangle -f^{*}(z)+g(u),$$

where f* denotes the convex conjugate of *f*. Under this set-up, PDHG can decouple the initial problem (2.1) into two simple problems, using as before the proximal operators of *g* and in addition the proximal operator of f*,

2.4 $${\mathrm{prox}}_{\tau f^{*}}(u):=\underset{v}{\text{arg min}}\frac{1}{2}||v-u|{|}_{2}^{2}+\tau f^{*}(v).$$

## 3. Case study I: colour image processing

**Figure 1.**
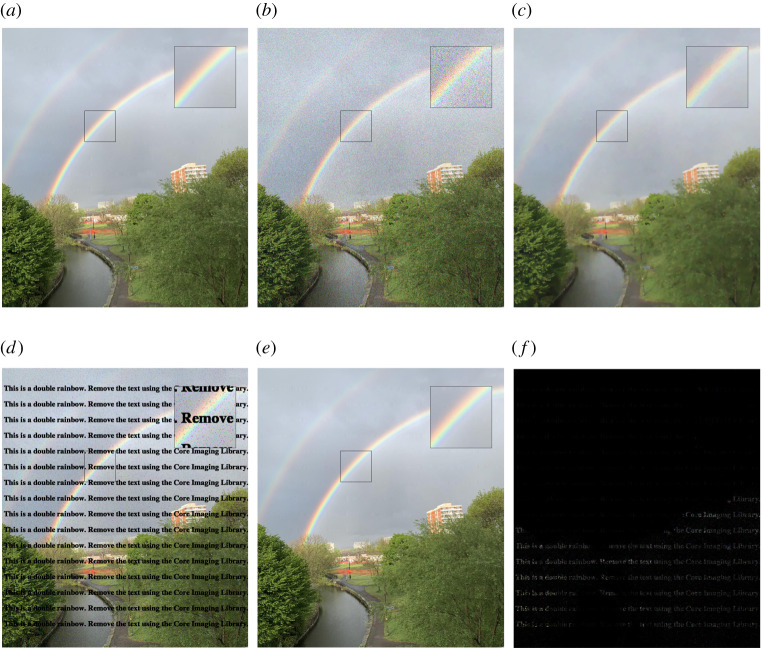
Colour processing: (1st row) total variation denoising. (2nd row) Total generalized variation inpainting and denoising. Regularizing parameters are optimized based on the SSIM value. (*a*) Ground truth, (*b*) noisy_data (Gaussian noise), (*c*) VTV denoising (*α* = 0.15) PSNR = 26.441, SSIM = 0.739, (*d*) noisy_data (salt and pepper noise with missing pixels), (*e*) TGV inpainting (*α*, *β*) = (0.5, 0.2) PSNR = 32.880, SSIM = 0.960, (*f* ) absolute difference |(*e*) − (*a*)|.

We begin our first demonstration with a case study within a colour imaging framework, i.e. a vector-valued image that has just three channels: red, green and blue. Our test data are a high resolution *double rainbow*^2^ image taken from a smartphone of 1194 × 1353 pixels and three channels, see figure 1*a*. We let

Ω={(i,j) |0≤i<M, 1≤j<N,M=1194, N=1353}

be a rectangular discretized grid representing our image domain and define an RGB colour image *u* as

$$u:\Omega \to R^{3},\;u=(u_{1},u_{2},u_{3}),$$

where $u_{k}\in R^{M\times N},\;k=1,2,3$ represent the red, green and blue channels. We consider the cases of

(a) denoising a noisy image corrupted by simulated Gaussian noise, see figure 1*b*,
(b) inpainting + denoising of a noisy image corrupted by simulated Salt & Pepper noise with missing text information, see figure 1*d*.

### (a) Colour denoising

We start with one of the most fundamental and well-studied problems in image processing, that is image denoising. In the pioneering work, Rudin, Osher and Fatemi (ROF), [19], introduced the TV regularizer to tackle image denoising for greyscale images u:Ω→R. Given a noisy image *b* corrupted by additive Gaussian noise, they solve the following optimization problem

3.1 $$u^{*}=\underset{u}{\text{arg min}}\frac{1}{2}||b-u|{|}_{2}^{2}+\alpha \mathrm{TV}(u),$$

where TV denotes the discretized TV defined as the ℓ^2,1^ norm of the gradient. If the gradient is *Du*: = (*D*_*y*_*u*, *D*_*x*_*u*), where *D*_*y*_, *D*_*x*_ denote the finite differences along the *y* and *x* directions, respectively, we write

3.2 $$\mathrm{TV}(u)=||Du|{|}_{2,1}=\sum_{i,j}^{M,N}{\left(|(D_{y}u,D_{x}u){|}_{2}\right)}_{i,j}=\sum_{i,j}^{M,N}{\left(\sqrt{{\left(D_{y}u\right)}^{2}+{\left(D_{x}u\right)}^{2}}\right)}_{i,j}.$$

The above definition can be extended for vector-valued images, which results in a vectorial version of the TV. The gradient for the RGB case is now *Du* = (*Du*_1_, *Du*_2_, *Du*_3_), where for each *k* = 1, 2, 3, *Du*_*k*_: = (*D*_*y*_*u*_*k*_, *D*_*x*_*u*_*k*_). The vectorial (channelwise) total variation (VTV), see [20] for more definitions of VTV, is defined as

3.3 $$\begin{array}{lll} \text{VTV}(u):=\|Du{\|}_{2,1} & = & \sum_{k=1}^{3}\sum_{i,j}^{M,N}{\left(|Du_{k}{|}_{2}\right)}_{i,j}\\ & = & \sum_{k=1}^{3}\sum_{i,j}^{M,N}{\left(\sqrt{{\left(D_{y}u_{k}\right)}^{2}+{\left(D_{x}u_{k}\right)}^{2}}\right)}_{i,j}. \end{array}$$

For both greyscale and coloured images, we can set up the regularizers (3.2) and (3.3) using the TotalVariation function in CIL. The optimization problem that we solve for the colour denoising is similar to (3.1) but using the VTV regularizer, i.e.

3.4 $$u^{*}=\underset{u}{\text{arg min}}\frac{1}{2}||b-u|{|}_{2}^{2}+\alpha \mathrm{VTV}(u),$$

where *b* is shown in figure 1*b*. One can observe that (3.4) is in fact the proximal operator (2.2) with *τ* = 1.0 evaluated at *b*. We solve (3.4), using the fast gradient projection (FGP) algorithm that is contained in the proximal method of the TotalVariation function.


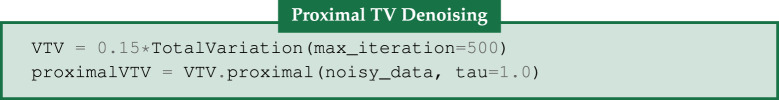


It is clear from figure 1 that noise is reduced, while preserving the edges of the image. However, TV is known for promoting piecewise constant reconstructions leading to images with blocky structures. This is called the staircasing effect and becomes apparent in smooth regions, see for instance the area around the rainbow in figure 1*c*.

### (b) Colour inpainting

Given an image where a specific region is unknown, the task of image inpainting is to recover the missing region from the known part of the image. A popular application of inpainting is in art restoration, where damaged or missing areas are repainted, i.e. filled, based on the surrounding context. We let D⊂Ω be a subdomain of Ω, i.e. the *inpainting domain*, where no data are known and missing information should be interpolated. In this example, our input image is shown in figure 1*d*, where in addition to Salt & Pepper noise, missing pixels from a repeated text are incorporated. A suitable data fidelity term for this kind of noise distribution is the *L*^1^ norm that acts on the domain Ω∖D.

To overcome the staircasing artefacts that TV promotes, we employ a higher-order regularizer, namely the total generalized variation introduced in [21]. We let *α*, *β* > 0 be regularization parameters and define

3.5 $${\mathrm{TGV}}_{\alpha ,\beta }(u)=\min_{w}\alpha ||Du-w|{|}_{2,1}+\beta ||Ew|{|}_{2,1},$$

where E denotes the symmetrized gradient operator defined as $Ew=(1/2)(Dw+Dw^{T})$. The optimization problem above provides a way of balancing between the first and second derivative of an image *u*. In particular, one expects that in the neighbourhood of edges, the second derivative *D*^2^*u* is relatively ‘large’, hence a reasonable choice is to let *w* = 0 in (3.5) and recover the TV regularizer. On the other hand, *D*^2^*u* is relatively small in smooth regions of an image and *w* = *Du* is a proper condition for the minimization problem (3.5). Under this format, edges are preserved, as in the TV regularizer, and additionally piecewise smooth structures are promoted.

The minimization problem under the TGV regularizer and the *L*^1^ norm as a data fidelity term is the following:

3.6 $$\begin{array}{rl} & u^{*}=\underset{u}{\text{arg min}}||Mu-b|{|}_{1}+{\mathrm{TGV}}_{\alpha ,\beta }(u)\Leftrightarrow \\ \text{and}\; & (u^{*},w^{*})=\underset{u,w}{\text{arg min}}||Mu-b|{|}_{1}+\alpha ||Du-w|{|}_{2,1}+\beta ||Ew|{|}_{2,1}, \end{array}\}$$

where the M is a diagonal operator with 1 in the diagonal elements corresponding to pixels in Ω∖D and 0 in D. In CIL, we use the MaskOperator that accepts as an input a two-dimensional boolean array, i.e. mask. Since we have a colour image, we employ the ChannelwiseOperator to encode the effect of missing pixels to the RGB channels. In order to solve (3.6), we use the PDHG algorithm, where the first step is to express (3.6) in the general form of (2.1). Let u=(u,w)∈X and define an operator K:X→Y as

3.7 $$K=\left[\begin{array}{cc} M & O\\ D & -I\\ O & E \end{array}\right]\Rightarrow Ku=K\left[\begin{array}{c} u\\ w \end{array}\right]=\left[\begin{array}{c} Mu\\ Du-w\\ Ew \end{array}\right]=\left[\begin{array}{c} y_{1}\\ y_{2}\\ y_{3} \end{array}\right]=y\in Y,$$

where O, I denote the zero and identity operators respectively. We continue with the definition of the functions *f* and *g*. The function *f* is a separable function that contains the three terms in (3.6) and is defined as

3.8 $$\begin{array}{rl} & f(y):=f(y_{1},y_{2},y_{3})=f_{1}(y_{1})+f_{2}(y_{2})+f_{3}(y_{3}),\text{ where}\\ \text{and}\; & f_{1}(y_{1}):=||y_{1}-b|{|}_{1},\;f_{2}(y_{2}):=\alpha ||y_{2}|{|}_{2,1},\;f_{3}(y_{3}):=\beta ||y_{3}|{|}_{2,1}, \end{array}\}$$

and *g*(***u***) = *g*(*u*, *w*) = *O*(*u*) ≡ 0 is the zero function.

Using (3.7) and (3.8), we have that

$$\begin{array}{rl} f(Ku)+g(u) & =f\left([\begin{array}{c} Mu\\ Du-w\\ Ew \end{array}]\right)=f_{1}(Mu)+f_{2}(Du-w)+f_{3}(Ew)\\ & =||Mu-b|{|}_{1}+\alpha ||Du-w|{|}_{2,1}+\beta ||Ew|{|}_{2,1}, \end{array}$$

which is exactly the objective function in (3.6).

In CIL, (3.7) can be expressed easily with the BlockOperator *K* and it is filled row-wise. The elements are the GradientOperator *D*, the IdentityOperator I, the SymmetrisedGradientOperator E, the ChannelwiseOperator M and two ZeroOperator O. The separable function in (3.8) can be expressed by the BlockFunction *f*, whose elements are the L1Norm, and two MixedL21Norm functions. Finally, *g* is the ZeroFunction function. We choose the PDHG algorithm to solve such an optimization problem. Without any user input, CIL will by default use primal/dual stepsizes *σ*, *τ* with *σ* = 1.0 and $\tau =1.0/\sigma ||K|{|}^{2}$ that satisfy $\sigma \tau ||K|{|}^{2}<1$ to guarantee convergence. We can monitor its convergence every 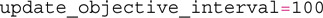 using 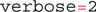. However, to speed up convergence it may be necessary to change such default values.


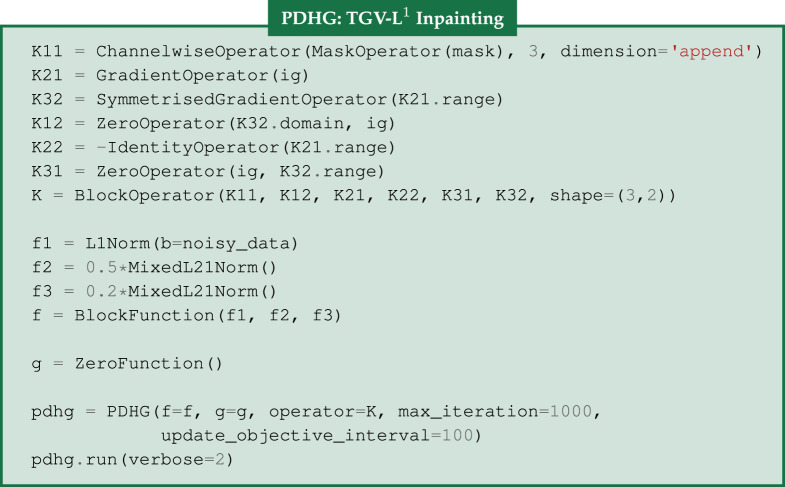


The TGV reconstruction is presented in figure 1*e*, where there are no staircasing issues and most of the repeated text is eliminated. We observe that the inpainting process behaves quite well when the background is relatively smooth, e.g. sky. However, in regions with specific textures, such as trees, leaves and grass, TGV inpainting could not completely restore the missing pixels, see the absolute difference between the ground truth and reconstruction in figure 1*f* . In the above two examples, the optimal regularization parameters are chosen to maximize the structural similarity index measure (SSIM), [22]. In terms of TGV, it is usually sufficient to find an optimal ratio *β*/*α*, thus reducing the number of parameters to be optimized, in order to obtain a high-quality reconstruction, [21,23].

How to select the best regularization parameter(s) is an important question and automated methods for doing this is an active research area beyond the scope of this article. This is of particular importance in real case scenarios when the comparison with the ground truth cannot guide this choice. In this paper, we choose to follow a direct grid search of parameters for simplicity. In a future release, we hope to provide reconstruction algorithms with automated parameter selection methods providing the user with an end-to-end pipeline. We refer the reader to the following papers where a number of methods to select the regularization parameters are presented for different imaging applications, e.g. the L-curve method [24,25], the S-curve method [26], the Morozov Discrepancy principle [27–30] and when the regularization parameter is spatially dependent, one can use [31–34].

## 4. Case study II: dynamic tomography

### (a) Motivation

The focus of this section is on dynamic CT, [35], where the aim is to scan a sample that undergoes some change, be it internal, such as an evolution of its composition or due to external input, like applied torque or compression, [36]. These changes must be slow with respect to the time it takes to acquire a single tomogram [37], otherwise the reconstructions would suffer from severe motion artifacts or the quantification would be meaningless. The duration of a CT scan is determined by the time needed to acquire a sufficient number of projections of the sample viewed from different angles with the required signal-to-noise ratio. This is determined mainly by detector performance and X-ray source intensity, and can vary from few projections per minute, as for laboratory X-ray CT scanners, to thousands of projections per second, as in the case of synchrotrons.

One way to increase the temporal resolution is by faster scanning through undersampling, i.e. by reducing the number of acquired projections, leading to sparse tomographic views. *Sparse CT* reconstruction is a highly ill-posed problem and has received great attention lately in the tomography community, [38–40] and especially in view of dynamic CT, [41–44]. Another beneficial consequence of Sparse CT is that it allows the reduction of radiation dose to the sample, which is for instance extremely useful in medical imaging where one can reduce both the radiation dose to the patient, and the duration of the imaging [45].

In the following, we focus on different reconstruction methods for sparse dynamic CT, using an open-access dynamic dataset available from [46]. The aim is to demonstrate how to increase the temporal resolution, or to reduce the radiation dose of a CT scan, without sacrificing the quality of the reconstructions. After the description of the dataset and of three possible undersampling configurations, we demonstrate how the standard reconstruction algorithm FBP, applied separately for each time step, leads to severe streak artifacts due to the limited number of projections. We then demonstrate how to use CIL to employ iterative reconstruction algorithms with three different regularization methods that incorporate prior information in the spatio-temporal domain to obtain quantitative information, to suppress the undersampling artifacts and noise on the reconstruction. At the end of the section, we compare the results obtained with all the reconstruction methods presented and demonstrate the improvements in temporal resolution and image quality enabled by a suitably chosen iterative reconstruction algorithm.

### (b) Data information

#### (i) Description

The sample was an agarose-gel phantom, [47], perfused with a liquid contrast agent in a 50 ml Falcon test tube (ø 29 × 115 mm). The aim of this experiment was to simulate diffusion of liquids inside plant stems, which cannot withstand high radiation doses from a denser set of measurement angles. After the agarose solidified, five intact plastic straws were made into the gel and filled with 20% sucrose solution to guarantee the diffusion by directing osmosis to the gel body.

#### (ii) Acquisition

Each scan was acquired in 4.5 min with intermissions of approximately 15 minutes between consecutive measurements. In total, the acquisition process lasted about 3 h leading to 17 sinograms, one for each time state. In addition, pre-scan and post-scan measurements are acquired with a noticeably higher number of projections; 720 and 1600 projections, respectively. The acquired sinograms are pre-processed using Lambert–Beer negative logarithm conversion.

#### (iii) Dataset

Every measurement consists of 360 projections with 282 detectors bins obtained from a flat-panel circular-scan cone-beam microCT-scanner. Only the central slice is provided, resulting in a *2D fanbeam geometry*. For this experiment, our reconstruction volume is 256 × 256 of 17 time frames. Additional metadata information, such as distance from source to the detector and distance from source to the origin are provided to set up the cone beam geometry.

### (c) Dynamic sparse CT set-up

**Figure 2.**
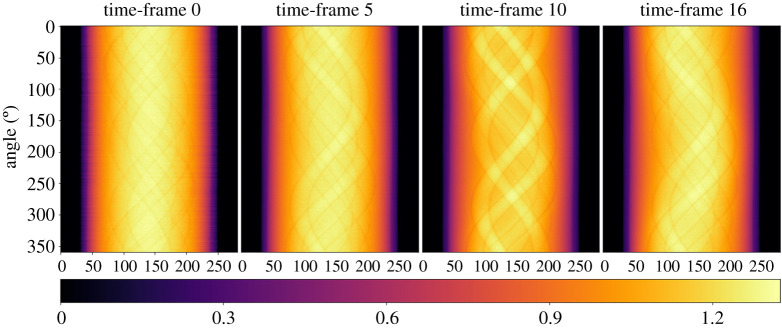
Sinogram data with 360 projection angles of the gel-phantom for four different time-frames.


Firstly, we configure our AcquisitionGeometry ag for a two-dimensional cone-beam geometry using information about the position of the source and the detector, the dimensions of the panel using set_panel and the projection angles using set_angles. In order to set up a multichannel geometry, we use set_channels which refer to the 17 time frames. Next, we allocate space for our acquisition dataset of 360 projection angles, data_360, that is filled with the corresponding sinograms for each of the 17 time frames. The Zenodo data are provided as a Matlab mat-file that can be read in e.g. using scipy.io.loadmat, which produces a list of sinograms containing the 17 sinograms, see figure 2. We obtain the default corresponding ImageGeometry ig for the acquired dataset of 360 projection angles using the get_ImageGeometry method from ag. The default dimensions are the same as the number of detector bins; here we reduce it to 256 by 256. Note that our image domain is a 2D + time spatiotemporal volume, i.e.

Ω={(i,j,t):0≤i<M,0≤j<N, 0≤t<T,M=N=256, T=17}.

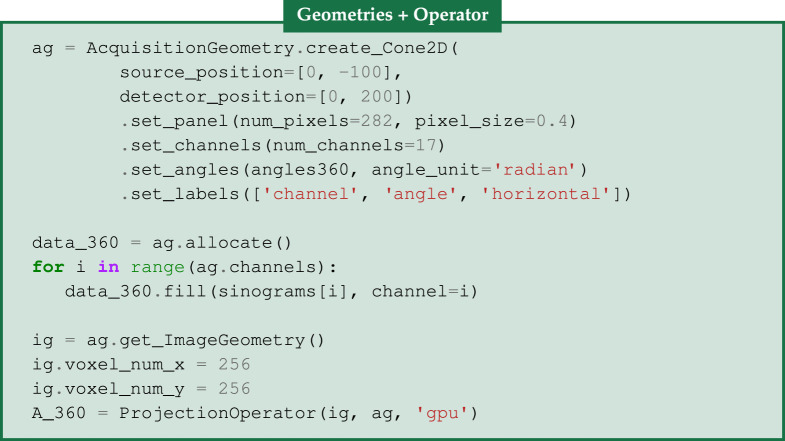


**Figure 4.**
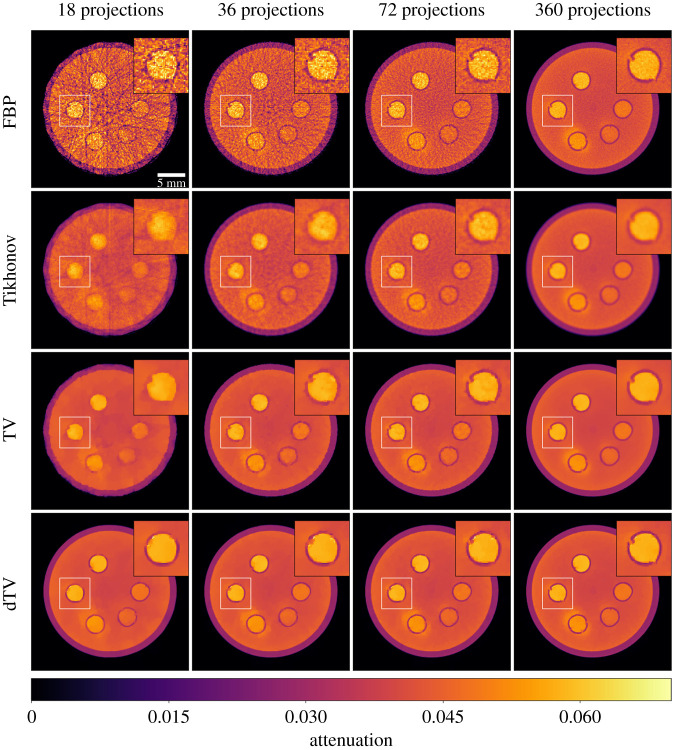
Tomographic reconstructions of the gel phantom, for the FBP algorithm, and regularization problems (4.1), (4.3), (4.6) with a different number of projections for the 8th time frame. The regularization parameters and PSNR/SSIM values are reported in table 1. All images share the same colour map.

We begin our reconstructions with the classical FBP algorithm with a Ram–Lak filter that is applied separately to the full data in each time frame, see top row in figure 4. In a practical sparse CT set-up, 360 projection angles would not be available, however, we use the full-data FBP reconstruction as our *ground truth* for assessing reconstruction quality from undersampled data quantitatively and for finding the optimal regularization parameters for the methods described below.

**Table 1.** PSNR and SSIM values averaged over all time frames for the FBP, Tikhonov (Tikh.), TV and dTV (*η* = 0.005) reconstructions at 18, 36 and 72 projections; highest scoring (in all cases dTV) in each column marked in italics. In addition the table lists optimal values of the regularization parameter *α* (in the sense of maximizing PSNR with respect to the ground truth).

	PSNR			SSIM			optimal *α* (10^−3^)		
Algo.	18	36	72	18	36	72	18	36	72
FBP	19.589	23.452	27.094	0.526	0.622	0.723	—	—	—
Tikh.	27.157	30.058	31.523	0.647	0.714	0.761	0.01	10	19
TV	28.719	31.903	32.919	0.720	0.765	*0.784*	0.72	0.54	0.81
dTV	*32.017*	*32.662*	*33.083*	*0.770*	*0.774*	*0.784*	8.1	7.2	8.1

In order to create undersampled dynamic data for different projection angles, we employ the Slicer processor, that is described in [4]. We create different equi-angular undersampling patterns, using the step sizes of [5, 10, 20], along the angle direction, leading to the datasets data_72, data_36, data_18 of 72, 36 and 18 projections, respectively. This means that we are able to increase the temporal resolution or reduce the radiation dose by a factor 5, 10 and 20, respectively. Finally, new projection operators are defined, e.g. A_72, A_36, A_18, based on the undersampled acquisition geometries and the image geometry that remains the same for all cases.


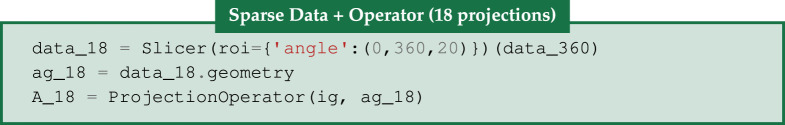


### (d) Tikhonov regularization

In [4], we presented in detail how one can set up Tikhonov regularization for single channel X-ray tomography. For our current dynamic case, we can formulate the identical problem, namely

4.1 $$u^{*}=\underset{u}{\text{arg min}}\;\frac{1}{2}||Au-b|{|}_{2}^{2}+\alpha ||Lu|{|}_{2}^{2},\;\text{(Tikhonov)}$$

where, *b* is now the multichannel sinogram, e.g. data_360 or data_18, containing all 17 time frame sinograms and *A* is now the corresponding multichannel projection operator, e.g. A_360 or A_18. The second term in (4.1) acts as a smooth regularizer, where the linear operator *L*, can be for example an identity or a gradient operator *D*, acting over the multichannel image data. In the case of *L* = *D*, we offer the user two different modes for the GradientOperator, where finite differences are computed only along the spatial dimensions or for both the spatial and channel dimensions. Therefore, if 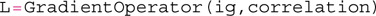, the derivatives across every direction in a three-dimensional volume are considered, i.e. *Du* = (*D*_*t*_*u*, *D*_*y*_*u*, *D*_*x*_*u*), if 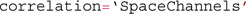, whereas if 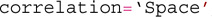, we take into account only the derivatives across the spatial dimensions, i.e. *Du* = (*D*_*y*_*u*, *D*_*x*_*u*). In the algorithm comparison, we demonstrate here finite differences over both space and channels, i.e. we use 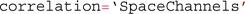. The code snippet to set up (4.1) in CIL is identical to the one presented in [4], hence it is omitted here.

### (e) Spatio-temporal TV

As a second regularization method, we apply an edge-preserving prior by replacing the *L*^2^ term in (4.1), with the TV regularizer, which in a spatio-temporal setting can be employed either in a channelwise fashion or as here over the full spatio-temporal volume, i.e.

4.2 $$\mathrm{TV}(u)=||Du|{|}_{2,1}=\sum_{i,j,t}^{M,N,T}{\left(\sqrt{{\left(D_{t}u\right)}^{2}+{\left(D_{y}u\right)}^{2}+{\left(D_{x}u\right)}^{2}}\right)}_{i,j,t}.$$

Under this isotropic coupling between space and time, the finite differences along the directions *t*, *y* and *x* are penalized equally with a single regularizing parameter, promoting piecewise constant structures in the spatio-temporal volume by solving

4.3 $$u^{*}=\underset{u\geq 0}{\text{arg min}}\;\frac{1}{2}||Au-b|{|}^{2}+\alpha \;\mathrm{TV}(u),\;\text{(spatio-temporal TV)}.$$

The above minimization problem can be solved using the (explicit) PDHG algorithm, [48], exactly as in the single-channel case as described in [4], decomposing it into two subproblems, where the two proximal operators, (2.2) and (2.4) have an explicit closed form solution. As in §3b, *f* is now a separable function, i.e. BlockFunction, containing the $||\cdot |{|}_{2}^{2}$, for the acquisition data, and $||\cdot |{|}_{2,1}$ norms. Consequently, we can express the operator *K* as a BlockOperator containing the multichannel ProjectionOperator *A* and the GradientOperator. Finally, to enforce a non-negativity constraint, we let *g* be the IndicatorBox with 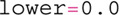. In the code snippet below, we define the triplet (*K*, *f*, *g*) used in PDHG for the case of 18 projections.


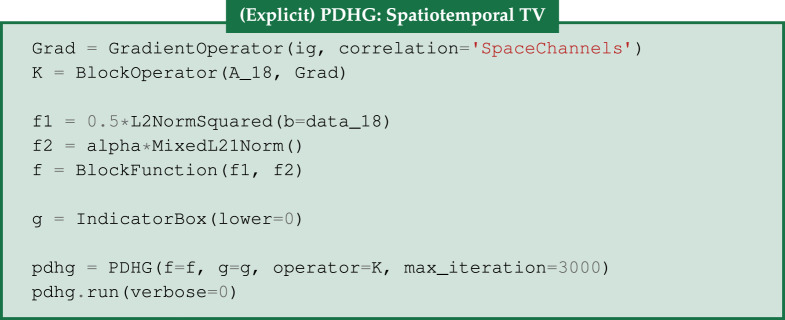


### (f) Directional TV

The third and final regularization method uses a structure-based prior, namely the dTV. To apply this variational method to sparse CT reconstruction, we adopt the framework of *parallel level sets* introduced in [49] and used for different applications such as multi-modal imaging, [50,51]. For example, an image from another modality, e.g. MRI, *reference image*, is known *a priori* and acts as additional information from which to propagate edge structures into the reconstruction process of another modality, e.g. PET. Another popular set-up is to use either both modalities or even channels in a joint reconstruction problem simultaneously, improving significantly the quality of the image, see for instance [52–54]. In a parallel level set framework, two images, *u* and *v*, are called structural similar if ∇u is parallel to ∇v, where *u* is the image to be reconstructed, given the known reference image *v*. In this sense, we are able to encode additional information on the location or direction of edges for the (*u*, *v*) pair. The dTV regularizer of the image *u* given the reference image *v* is defined as

4.4 $$d\mathrm{TV}(u,v):=||D_{v}\nabla u|{|}_{2,1}=\sum_{i,j=1}^{M,N}{\left(|D_{v}\nabla u{|}_{2}\right)}_{i,j},$$

where the weight *D*_*v*_ depends on the normalized gradient *ξ*_*v*_ of the reference image *v*,

4.5 $$D_{v}=I_{2\times 2}-\xi_{v}\xi_{v}^{T},\;\xi_{v}=\frac{\nabla v}{\sqrt{\eta^{2}+|\nabla v{|}_{2}^{2}}},\;\eta >0.$$

The vector-field *ξ*_*v*_ is able to capture structural information from the reference image depending on the edge parameter *η* and determine which directions to be penalized. For instance, we have that $D_{v}\nabla =(1-|\xi_{v}{|}_{2}^{2})\nabla$, if ∇u||∇v. Equivalently, if |*ξ*_*v*_|_2_ > 0, aligned gradients are favoured. On the other hand, if ∇u⊥∇v then $D_{v}\nabla =\nabla$. Finally, note that 0 ≤ |*ξ*_*v*_|_2_ < 1, where the lower bound is attained for $|\nabla v{|}_{2}=0$ (constant regions) and the upper bound when $|\nabla v{|}_{2}\to \infty$ (edges).

**Figure 3.**
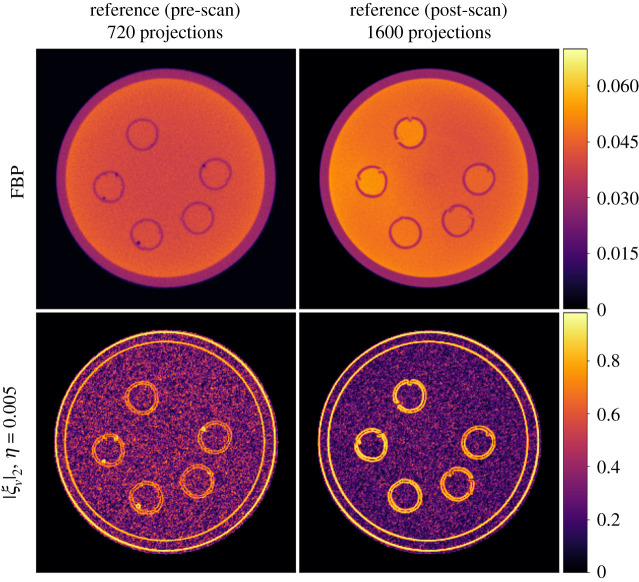
FBP reconstructions for the acquired data (pre/post scans) with 720 and 1600 projections used as reference images for the dTV regularizer. The normalized gradient *ξ*_*v*_ for edge parameter *η* = 0.005. In both cases, the edges are clearly seen in the |*ξ*_*v*_|_2_ images. In the latter case, where noise is lower due to the larger number of projections, the edge promoting effect of dTV can be expected to be stronger.

In figure 3, we show the pre- and post-scan FBP reconstructions acting as the reference images, along with |*ξ*_*v*_|_2_ that illustrates how edge information is captured by *ξ*_*v*_ to be included by the dTV regularizer. For each time frame *t*, we solve the following problem

4.6 $$u_{t}^{*}=\underset{u_{t}\geq 0}{\text{arg min}}\;\frac{1}{2}||A_{\text{sc}}u_{t}-b_{t}|{|}^{2}+\alpha \;d\mathrm{TV}(u_{t},v_{t})\;\text{(dynamic dTV)},$$

where *A*_sc_, *b*_*t*_, u*_t_, denote the single channel ProjectionOperator, the sinogram data and the reconstructed image for the time frame *t* respectively. In terms of the reference images ${\left(v_{t}\right)}_{t=0}^{T-1}$, we use $v_{0}=v_{\text{pre\_scan}}$, i.e. the FBP reconstruction of the pre-scan data with 720 projections, and $v_{t}=v_{\text{post\_scan}}$, *t* = 1, …, *T* − 1, for the FBP reconstruction for the data with 1600 projections. We follow this configuration, because we notice a slight movement of the sample at the beginning of the experiment. One could apply other configurations for the reference image in the intermediate time frames. For example, in order to reconstruct the (*t* + 1)th time frame, one could use the *t*th time frame reconstruction as reference. A more sophisticated reference selection approach is applied in hyperspectral computed tomography in [54].

Similarly to (4.3), we solve (4.6) using the PDHG algorithm, but with an alternative setup for the triplet (*K*, *f*, *g*). This time one of the subproblems is not solved explicitly but an inner iterative solver is used, known as implicit PDHG, see [55]. In particular, we let *g* be the FGP_dTV regularizing Function from **cil.plugins.ccpi_regularization** module of CIL, which wraps a GPU-accelerated implementation of the FGP algorithm in the CCPi-RGL toolkit. Since each time frame is solved independently of the others, the operator *K* is now the projection operator for a single channel K_sc and the functions *f* and *f*_0_ are $||\cdot |{|}_{2}^{2}$ norms. To store the two-dimensional reconstruction for every time frame, we use the variable solution allocating space from the all-channel image geometry ig. Then, we use the fill method to store the reconstruction of every time step to the solution.


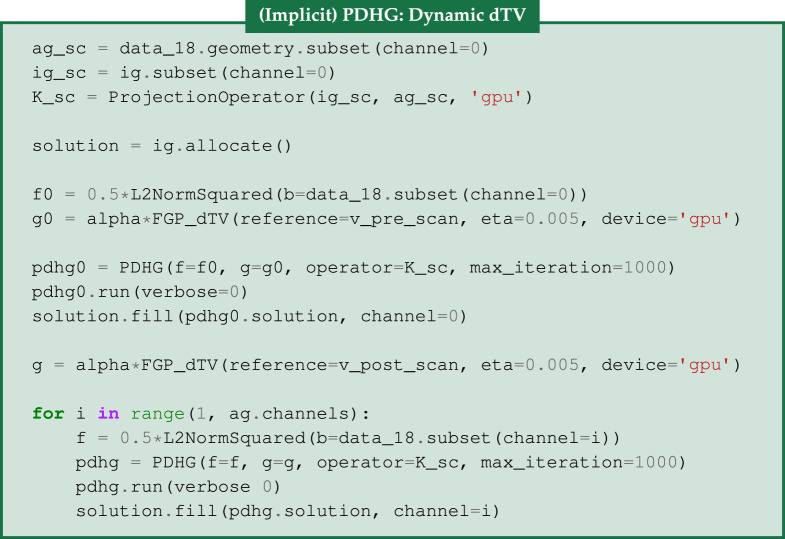


For the FGP_dTV Function, we need to specify the corresponding reference image, the regularization parameter and the smoothing parameter *η* appeared in (4.4). The optimal parameters *α* and *η* are reported in table 1. In the above code block, we define the projection operator, the image and acquisition geometries for the case of 18 projections. Then, the functions *f*_0_, *g*_0_ are used in the PDHG algorithm to reconstruct the first time frame using the v_pre_scan as a reference and the functions *f*, *g* concern all the other frames using the v_post_scan as a reference.

### (g) Results

**Figure 5.**
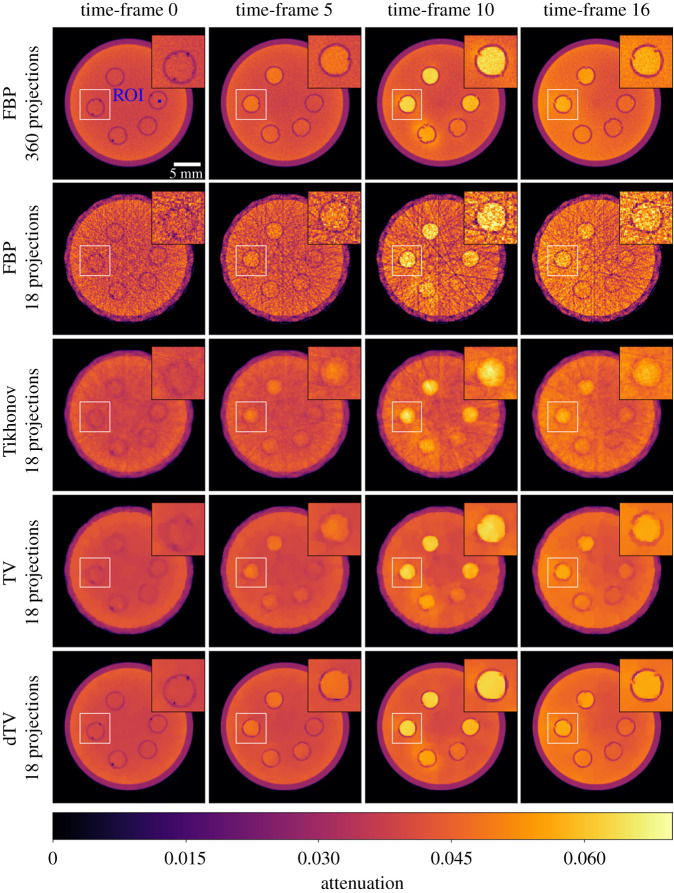
Tomographic reconstructions of the gel phantom, for the FBP algorithm with 360 and 18 projections, and for the regularization problems (4.1), (4.3), (4.6) with 18 projections for four different time frames. All images share the same colour map.

In this section, we present results for all the reconstruction methods presented above, e.g. channelwise FBP algorithm, Tikhonov, TV and dTV regularizations. In figure 4, we present a static comparison for all the reconstructions, for three undersampled data with 18, 36 and 72 projections as well as the full 360 projections for the 8th time frame. In figure 5, we provide a temporal comparison for four different frames, for the most interesting case of 18 projections, as it provides the greatest reduction in acquisition time.

Although, FBP produces satisfying reconstructions for 360 projections, reducing the number of projections results in streaking artifacts and a decrease in signal-to-noise ratio which is more pronounced the higher the undersampling, see first row of figure 4. Moreover, the quantification of the dynamic process is severely hindered by the undersampling, see for instance the first two rows of figure 5, showing the FBP reconstructions for 360 and 18 projections for four different time frames.

Compared to the FBP reconstructions, we observe that Tikhonov regularization can suppress both the noise and the streak artefacts, especially for a very low number of projections, see second row of figure 4. However, due to the *L*^2^ penalty term appearing in (4.1), edges as well as small details of the image are oversmoothed. In the third row of figure 4, we observe that noise is completely eliminated by the TV regularization and edges are preserved around the five circular cavities. This is more obvious in the cases of 72 and 360 projections. For fewer numbers of projections, staircasing artefacts, introduced by TV, are more apparent both spatially and temporally, see the blocky artifacts outside the boundaries of these cavities in figure 5. In addition, due to the low iodine concentration level at the earlier stage, see time frames 0 and 5 in figure 5, we witness a significant loss of contrast, particularly for the case of 18 projections.

In terms of the dynamic dTV reconstructions, we observe a significant contrast improvement for all the undersampled data, see the interior of the five cavities. Furthermore, edges are now well preserved for all the time frames due to the structural information that is integrated from the reference images $v_{\text{pre\_scan}}$ and $v_{\text{post\_scan}}$. For instance, we note sharper boundaries around the cavities compared to the TV reconstructions, specially for the lowest number of projections. This is also evident for different time frames for the 18 projections case, as one can see in the last row of figure 5, where overall dTV produces the best reconstructions. In particular, we note how the outer cylindrical edge is correctly reproduced as circular by dTV at 18 projections unlike all other methods which produce a polygonal outer edge due to the low number of projections.

**Figure 6.**
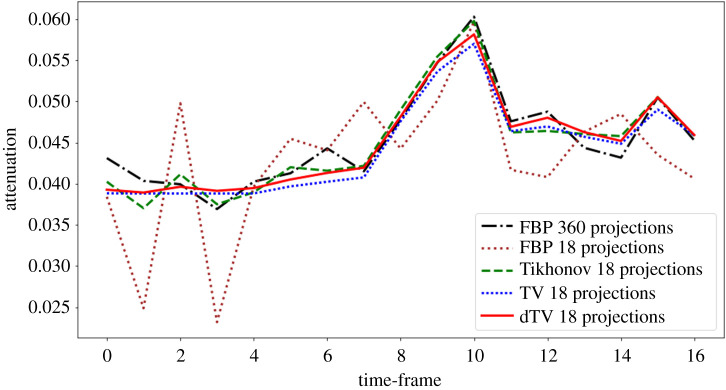
Temporal variations in the attenuation of the gel-phantom for specific ROI (blue) shown in the top left reconstruction of figure 4.

In figure 6, we compare the time activity (i.e. the reconstructed attenuation value over time) of the ground truth with the Tikhonov, TV and dTV reconstructions with 18 projections, for the single-pixel ROI appeared in the left image of figure 5. As expected, we observe very high oscillations for the FBP reconstruction with 18 projections, which can be reduced using Tikhonov regularization. Since there is no remarkable temporal variation until the 8th frame, we observe an almost similar behaviour for the TV and dTV reconstructions. However, dTV reconstruction provides a better contrast compared to the TV reconstruction that is more apparent after the 9th frame. Overall both dTV and TV are able to reproduce at 18 projections the single-pixel centre-of-cavity time activity at the same (or even better) quality as FBP using the full 360 projections.

**Figure 7.**
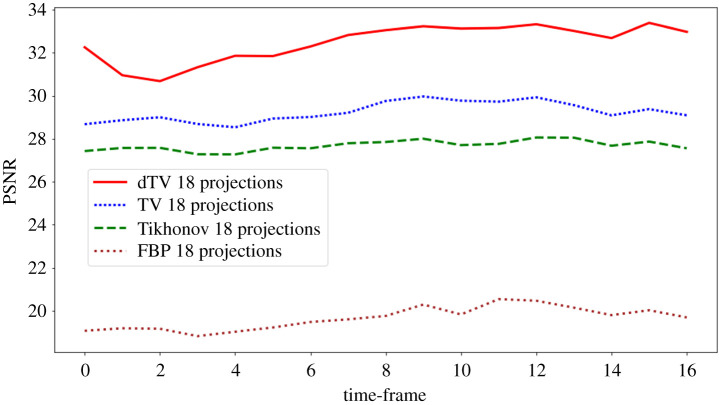
PSNR values per time frame for the FBP, Tikhonov, TV and dTV reconstructions with 18 projections, compared with *ground truth.*

In the problems (4.1), (4.3) and (4.6), all the corresponding regularization parameters are optimized based on the highest average of PSNR values over all the time frames compared to ground truth. In figure 7, PSNR values per time frame are computed for the FBP, Tikhonov, TV and dTV reconstructions for 18 projections with respect to the ground truth. Overall, dTV has the highest PSNR for all time steps, followed by the TV and Tikhonov regularizations and finally FBP reconstruction. We also report the PSNR and SSIM values for all cases of undersampled data and the optimized parameters *α*, *η* for all the regularization methods. We observe that for the very limited angular cases, e.g. (18, 36), dTV reconstructions produce better results, whereas increasing the number of projections dTV and TV reconstructions are comparable.

### (h) Discussion and conclusion

In conclusion, we have described three multi-channel regularized reconstruction methods for reducing undersampling artifacts in sparse Dynamic CT and their implementation using the modular building blocks of CIL. We conducted a comparative study of algorithm performance at three different undersampling levels of the full dataset which simulated an increase of the temporal resolution of the acquisition by a factor of 5, 10 and 20, respectively. The results demonstrated that the dTV method in particular is capable of obtaining high-quality reconstructions from reduced data and using it one can obtain the same quantitative information at a factor of 20 undersampling compared to channelwise FBP applied to full data. It is worth noting that for the dTV method of §4f access to high-quality pre- and post-scans is required. We want to stress that the design of the acquisition is crucial. For instance, should the experiment be acquired using a golden-ratio angular sampling scheme, [37], the experiment could have been let to run continuously and the time frame separation could have been decided as a post-processing step. In such a case, figure 6 could have shown a temporal resolution of up to 340 = 17 × 20 time points, with 18 angles per tomography time frame dataset.

## 5. Case study III: hyperspectral tomography

### (a) Motivation

For our final case study, we focus on hyperspectral X-ray CT imaging and how CIL can provide tools to reconstruct and analyse the internal elemental chemistry of an object. In every pixel, hyperspectral photon-counting detectors can measure the deposited energy during a certain exposure time and consequently calculate the associated photon energy of that pixel in that frame. This is repeated many times during the scan, where finally all events are binned into a single spectrum per pixel. Moreover, these types of detectors can achieve a high energy resolution (typically less than 1 keV) and can image over hundreds of spectral bands, and allow us to distinguish materials based on the element characteristic absorption edges, i.e. K-edges.

The main goal of this study is to identify elements of gold and lead from a mineralized ore sample from a goldrich hydrothermal vein. These materials (Au and Pb) typically appear in very low concentrations, with deposit size similar to our reconstructed voxel size. With no *a priori* knowledge of the distribution of these materials, and the inherently low-count hyperspectral data it is difficult to achieve satisfactory reconstruction with conventional methods. We demonstrate here how CIL can be used to easily implement a number of bespoke multi-channel regularized reconstruction methods in order to accurately identify and segment these deposits.

In the following, we describe the dataset, then we propose and compare three different reconstruction methods. First, we consider the standard SIRT algorithm which does not make use of any prior information, along with a variant in which the reconstruction of channel *i* is used to warm-start reconstruction of channel *i* + 1. Next, we describe two advanced regularization techniques to reconstruct four-dimensional hyperspectral data with different correlation between spatial and energy information. Finally, we describe how the Stochastic PDHG (SPDHG) algorithm, [10,56] that uses only a subset of the whole data in every iteration, can be used to accelerate computations of large dataset reconstruction.

### (b) Data information

#### (i) Description

The sample (ø 20 mm) is extracted from a hydrothermal vein from the Leopard Mine, Silobela, Zimbabwe. It contains materials such as pyrite (FeS_2_), quartz (SiO_2_), gold (Au) and minor amounts of galena (PbS), chalcopyrite (CuFeS_2_) and bornite (Cu_5_FeS_4_).

#### (ii) Acquisition

For the data acquisition, the authors, [57], use a colour imaging bay, in the Manchester X-Ray Imaging Facility (www.mxif.manchester.ac.uk). It is designed to be a flexible work bench for spectroscopic X-ray imaging and tomography. The corresponding detector is a High Energy X-ray imaging Technology (HEXITEC) spectroscopic detector that is installed in a Nikon XTH 225 system. The sample is scanned in cone-geometry setup along five separate horizontal positions for increased field of view. Each sub-projection was acquired with exposure time of 45 s (that is 225 s for a full stitched projection) with 120 projections covering $360^{\circ }$. The total scan time was 7.5 h. The acquired four-dimensional raw sinogram data has three spatial dimensions and one spectral dimension, i.e. 120 projection angles of 80 × 400 pixels with 800 energy bins ranging from 1.82 keV to 186.07 keV. The reconstruction volume will be 400 by 400 by 80 voxels.

#### (iii) Dataset

**Figure 8.**
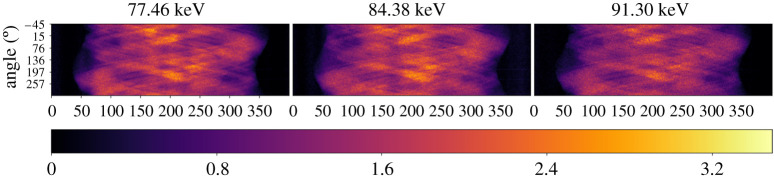
Selected sinograms at three different energies from hyperspectral X-ray CT dataset consisting of 800 energy channels, 120 projection angles and 400 detector bins; sinogram for the 20th vertical slice out of a total 80 slices.

All the files for this study are freely available and can be downloaded from [58]. It contains (a) 4D hyperspectral (energy-resolved) X-ray CT projection data, (b) flat-field data, (c) energy in keV for every energy bin and (d) geometric metadata of the cone-beam setup. Sinogram data from selected energy channels are presented in figure 8. These have been pre-processed by taking the natural logarithm of the normalized intensity in every spectral band and corrected for severe vertical stripes that would cause ring artifacts, using the RingRemover processor. During the acquisition process 800 energy channels were recorded. It is possible to consider the full energy range, however for demonstration purposes, we choose to examine an energy interval, [75.15 keV, 93.37 keV], of 80 channels that encompasses the K-edges of gold (Au, 80.725 keV) and lead (Pb, 88.005 keV).

### (c) SIRT and warm-started SIRT reconstruction

For our first reconstruction method, we use the SIRT algorithm, an algebraic iterative method for a particular weighted least-squares problem which in addition accepts certain convex constraints such as a non-negativity constraint, see [4]. We enforce this as in §4e with the IndicatorBox function. SIRT is applied channelwise for each three-dimensional dataset.

In addition to channelwise SIRT, one can enable a warm initialization, that allows a basic form of channel correlation in the reconstruction as used in [57]. Here, we initialize the SIRT algorithm for the (*i* + 1)th channel with the solution of the *i*th channel.


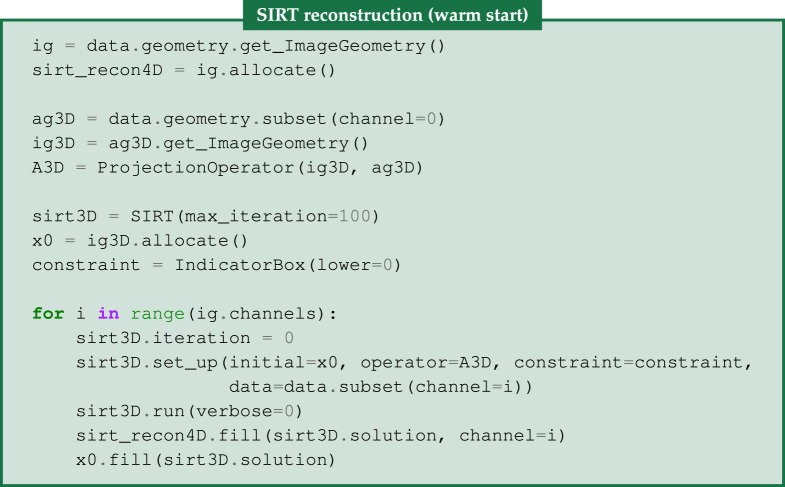


As shown in the code, we first need to extract the acquisition and image geometries, i.e. ig3D and ag3D, respectively, using the geometry information for one of the channels of the four-dimensional data. Then, we set up the corresponding single-channel projection operator A3D. For every channel, we run 100 iterations of the sirt3D instance of the SIRT algorithm and its solution is filled to the corresponding channel i of the final four-dimensional reconstruction sirt_recon4D.

### (d) Spatiospectral TV & (3D + spectral) TV regularization

To be able to preserve edges both in the spatial domain and absorption K-edges in the energy spectrum we propose to use the TV regularization, subject to a non-negativity constraint. We first consider the TV regularizer extended to a four-dimensional volume, where the gradient *Du* = (*D*_*e*_*u*, *D*_*z*_*u*, *D*_*y*_*u*, *D*_*x*_*u*) is coupled isotropically. We solve

5.1 $$u^{*}=\underset{u\geq 0}{\text{arg min}}\frac{1}{2}||Au-b|{|}_{2}^{2}+\alpha ||(D_{e}u,D_{z}u,D_{y}u,D_{x}u)|{|}_{2,1}\;\text{(spatio-spectral TV)}.$$

This regularizer combines the spatial and energy variations which are penalized by a single regularizing parameter. However, this may not be a good choice as the magnitude of the gradient in the spectral dimension will not necessarily be of the same order of magnitude as the one on the spatial dimensions. Therefore, it may be better to enforce separate regularization with respect to the energy and spatial gradients, i.e. *D*_*e*_*u* and (*D*_*z*_*u*, *D*_*y*_*u*, *D*_*x*_*u*), respectively. We therefore consider an alternative formulation with separate decoupled TV regularizers for the spectral and spatial dimensions, i.e.

5.2 $$u^{*}=\underset{u\geq 0}{\text{arg min}}\frac{1}{2}||Au-b|{|}_{2}^{2}+\beta ||D_{e}u|{|}_{1}+\alpha ||(D_{z}u,D_{y}u,D_{x}u)|{|}_{2,1}\;\text{(3D + spectral)TV}.$$

One can solve the above problems using for instance the (explicit) PDHG algorithm. For the triplets (*K*, *f*, *g*), the function *g* is the same for both problems (IndicatorBox) and the difference is with respect to the operator *K* and the functions *f*. In (5.1), the operator *K* and function *f* are similar to the (explicit) PDHG algorithm described in the dynamic CT section. To achieve the separation of the spatial and energy components of the GradientOperator, we can set the parameter 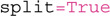 so that it will split the spatial gradients and the gradient along the energy direction, i.e. (*D*_*e*_, (*D*_*z*_, *D*_*y*_, *D*_*x*_)). Similarly, we need to provide a decomposition for the function *f*, using two BlockFunction that contain the three terms presented in (5.1). The following code blocks present the definition of the triplets (*K*, *f*, *g*) for the problems (5.1), (5.2), required to run the PDHG algorithm as described in the previous sections.


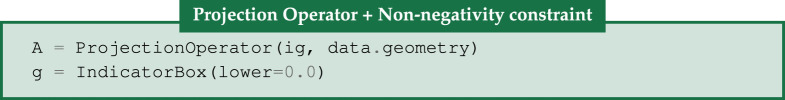



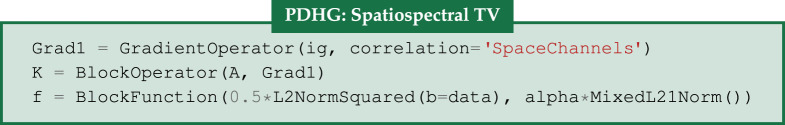



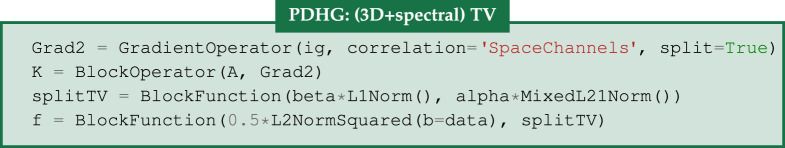


### (e) SPDHG algorithm

Although we can follow exactly the same set-up presented in the previous section to solve the above problems, one has to perform forward and backward operations of the projection operator *A* for the whole multichannel dataset every iteration. These operations are computationally expensive, especially for large datasets. In order to overcome this problem, CIL allows the user to employ the stochastic PDHG (SPDHG) algorithm, where the above operations are applied to a randomly selected subset of the data in every iteration. SPDHG has been used for different clinical imaging applications, such as PET, [56] and motion estimation/correction in PET/MR, [15], and produces significant computational improvements over the PDHG algorithm.


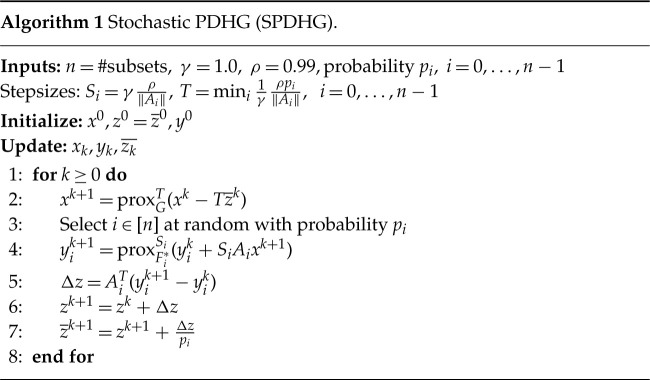


The set-up of the SPDHG algorithm is similar to the PDHG algorithm with the notable differences that we need to define the subsets, which are the *n* terms in the sum (2.1), as well as a list of probabilities for each subset to be selected in every iteration, see algorithm (e) for SPDHG pseudocode. SPDHG, as well as PDHG, can be set up in an explicit form, when the regularizer is a term in the sum, or in the implicit form where it is represented by *g*.

In our hyperspectral reconstruction, we use the explicit form of SPDHG. The 120 projections which constitute the acquisition data are split into *S* = 10 *data subsets* of 12 angles each, $b={\left(b_{i}\right)}_{i=0}^{S-1}$. In addition, we will have one *regularizer subset*. We can now rewrite (5.1) and (5.2), in the form (2.1) where *n* = *S* + 1 and *A*_*i*_ represents the projection operator for a data subset, *b*_*i*_, and with $f_{i}=0.5||A_{i}u-b_{i}|{|}_{2}^{2}$ for *i* = 0, …, *S* − 1, and $A_{n-1}=\nabla$ with *f*_*n*−1_ being the 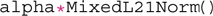 for the spatio-spectral TV regularizer or the splitTV for the (3D + spectral) TV regularizer.

We can configure different sampling patterns, i.e. the choice of the probabilities *p*_*i*_ to select the *i*th term of (2.1). One may choose to assign an equal probability for every term *n*, i.e. *p*_*i*_ = 1/*n*, *i* = 0, …, *n* − 1; we refer to this as *uniform sampling*. Another option, known as *balanced sampling*, is to give a probability 0.5 to select the regularizer subset and 0.5/*S* to select any one *data subset*. In the following, we choose the balanced sampling approach and refer the reader to [56] for a discussion on SPDHG sampling patterns.

We use the Slicer processor to obtain the data subsets ${\left(b_{i}\right)}_{i=0}^{S-1}$. For each data subset’s acquisition geometry, we create a list of operators (*A*_0_, …, *A*_*S*−1_), using the ProjectionOperator that share the same image geometry ig. For the function *f*, we use a list of the L2NormSquared Function with respect to each of the data subsets *b*_*i*_ for every *i* = 0, …, *S* − 1.


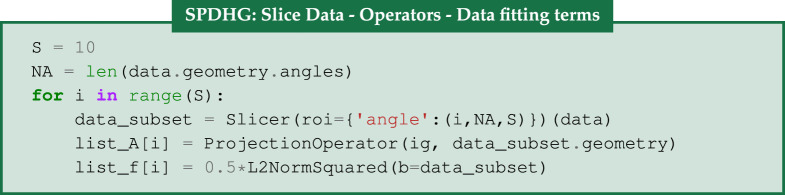


Depending on which problem we solve, the corresponding gradient operator is appended to the list of (*A*_*i*_) operators, i.e. Grad1 or Grad2 and wrapped using the BlockOperator. Similarly, the list of data fidelity terms are wrapped as a BlockFunction *f*.


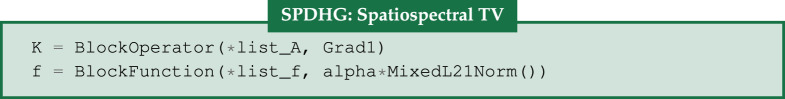



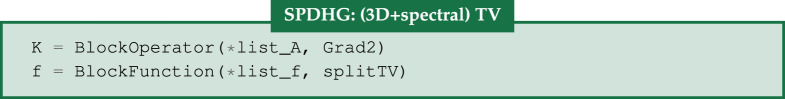


Finally, the code to set up and run the SPDHG algorithm for both (5.1), (5.2) minimization problems under a non-negativity constraint and using a list of subset probabilities specifying balanced sampling is:


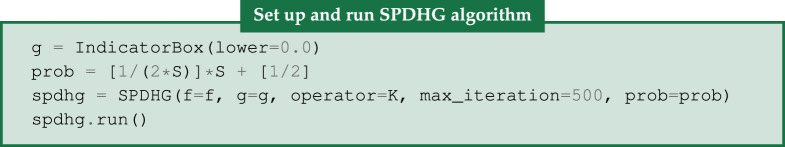


### (f) Results

**Figure 9.**
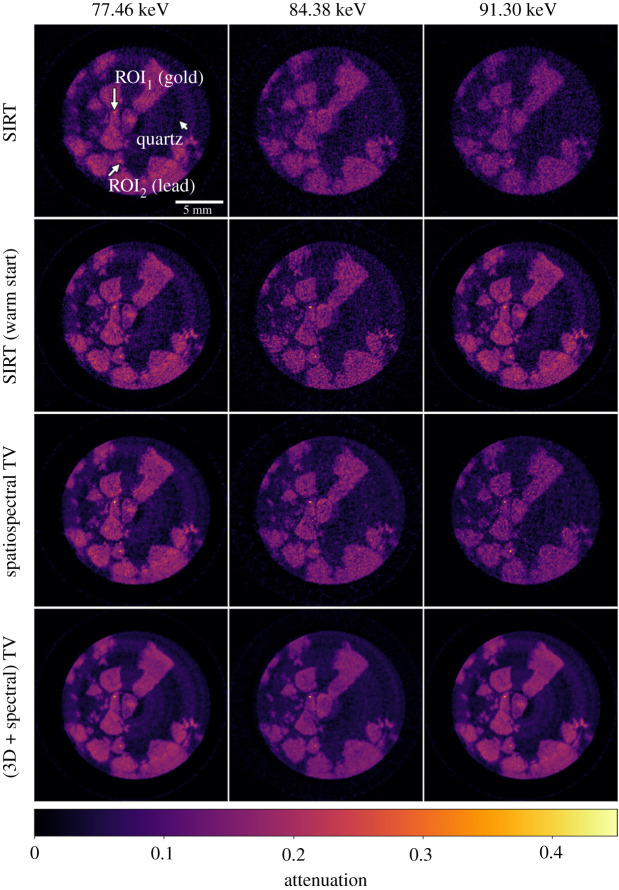
SIRT reconstructions without and with channel correlation, Spatiospectral TV and (3D + spectral) TV reconstructions using the SPDHG algorithm. Three different energies are presented at the 20th vertical slice: (1st column) below the gold K-edge, (2nd) after the gold K-edge but before the lead K-edge and (3rd) above the lead K-edge.

**Figure 10.**
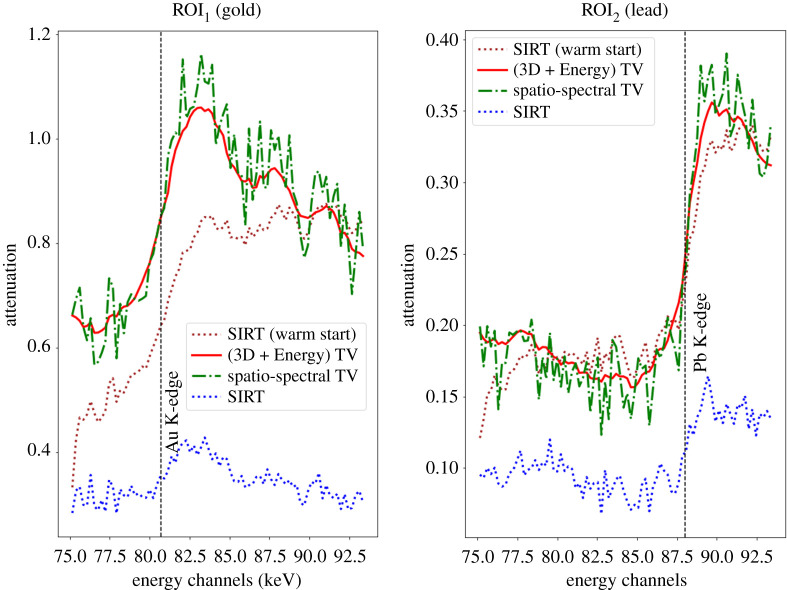
Attenuation plots as a function of X-ray energy for SIRT (without and with channel correlation), Spatio-spectral TV and (3D + spectral) TV reconstructions for the gold (ROI_1_) and lead (ROI_2_) shown in figure 9.

To assess performance of the algorithms considered, we reconstruct the hyperspectral dataset and compare reconstructions visually (figure 9) and in terms of their ability to reproduce the expected sharp K-edge jumps in gold and lead containing voxels (figure 10).

In the first rows of figure 9, we present results for the two versions of SIRT at three different energies, below, in between and above the K-edges of Au at 80.725 keV and Pb at 88.005 keV. Since in the basic SIRT algorithm there is no regularization to remove the noise, it is difficult to locate the ROIs of gold and lead for low energies, see the first row in figure 9. When the channels are linked by warm-started SIRT, we observe better contrast on these specific ROIs and both gold and lead materials are easy to distinguish. However, due to high spectral noise, the SIRT energy plots show highly oscillatory attenuation profiles, particularly for the Au plot, see figure 10. This could make the detection of the K-edge and hence the material unreliable, particularly for cases where we do not have prior knowledge of elemental composition.

In the last rows of figure 9, we present the spatio-spectral TV and (3D + spectral) TV reconstructions for three different energy channels, using the SPDHG algorithm with 10 subsets and 25 epochs that correspond to 500 iterations. The regularization parameters *α* are chosen by visual comparison reducing noise and preserving edges both spatially and along the spectral direction. Compared to the SIRT reconstructions, noise is reduced spatially and contrast is enhanced using the spatio-spectral TV regularizer. However, moving along different channels noise is still apparent, see the first row in figure 9 and green energy curves in figure 10. This is because the spectral differences have less impact compared to the spatial differences in the isotropic coupling of (5.1). However, the spectral noise could be reduced further by choosing a higher regularizing parameter for the spatio-spectral TV, but then small features would be lost spatially due to loss of contrast. This is an inherent limitation of the coupled spatio-spectral regularization. On the contrary, in the decoupled spatial and spectral regularization approach of the (3D + spectral) TV we have the freedom to balance the strength between space and spectral directions by suitable choices of the parameters *α* and *β*. In this way with the (3D + spectral) TV, we obtain higher quality reconstructions with better contrast and less noise, as seen in bottom row of figure 9 and red energy curve of figure 10.

### (g) PDHG versus SPDHG

**Figure 11.**
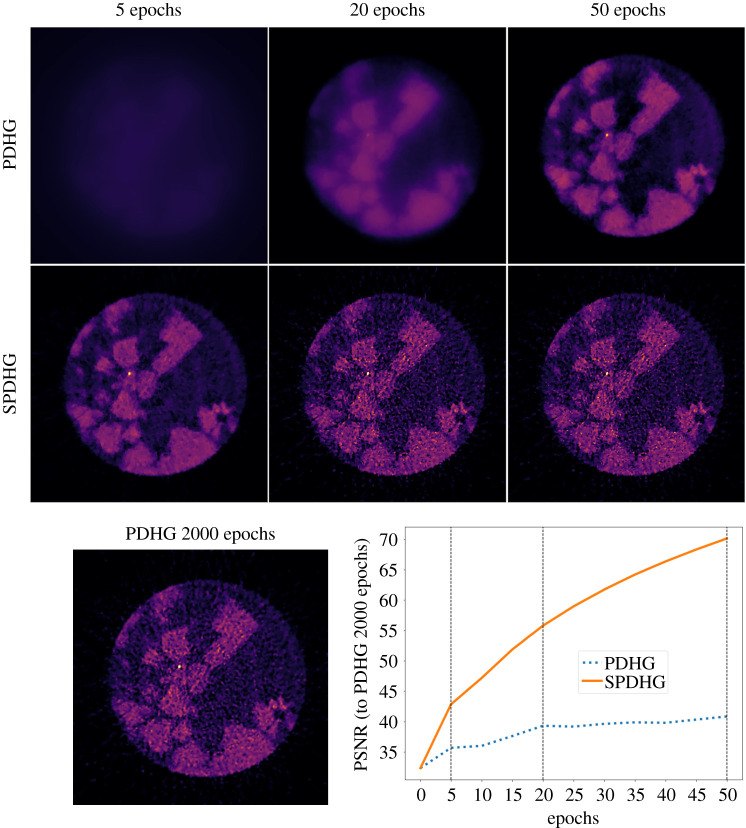
PDHG and SPDHG reconstructions of (5.1) with *α* = 0.001 for different epochs using only five channels and five vertical slices. PSNR comparison of the PDHG and SPDHG reconstructions with respect to the PDHG reconstructions after 2000 iterations. Energy channel at 84.38 keV for the 20th vertical slice is presented.

In figure 11, we demonstrate the computational benefit of SPDHG compared to the PDHG algorithm. On a cropped four-dimensional dataset with only five channels and five vertical slices, we present the spatio-spectral TV reconstructions for these algorithms with respect to the number of epochs. One epoch is the expected number of iterations for the algorithm to have processed all the data, i.e. all data subsets once. For PDHG, the full data are used in each iteration, so an epoch here equals an iteration. On the other hand, for SPDHG an epoch is determined by the number of data subsets. In our case, we use *S* = 10 data subsets with balanced sampling, which means that on average half the iterations call the regularizer and in the other half one of the data subsets is chosen with uniform probability. Hence, 20 iterations are required on average to process all the 10 data subsets, so an epoch for SPDHG equals 20 iterations.

We run PDHG for 2000 iterations = epochs and SPDHG for 1000 iterations = 50 epochs. We observe that even after five epochs, a meaningful reconstruction is obtained using the SPDHG algorithm, whereas in PDHG no structures of the rock are observed. In fact, the SPDHG reconstruction after five epochs is visually closer to the PDHG reconstruction after 50 epochs. This is also verified by the PSNR plot in figure 11. There, we compute for every epoch, the PSNR of the SPHDG and PDHG reconstructions against the PDHG reconstruction after 2000 epochs, which is considered as the reference image u*, that is the converged solution of equation (4.2).

A discussion of the computational advantages of the SPDHG versus the PDHG algorithm in tomography applications is beyond the scope of this article. Our intention was merely to demonstrate the CIL implementation of the SPDHG algorithm, which allows researchers to experiment with accelerated reconstruction of their own problems of interest.

### (h) Discussion and conclusion

In this particular rock sample, absorption K-edges are well defined and identifying the elements from these abrupt changes on the spectrum is relatively easy. However, this is not always the case, for instance, if there is no prior knowledge of the sample composition, or low chemical concentration and the sample deposits are on the order of detector voxel size. In such cases, K-edges may be completely concealed by the background noise of a channelwise FBP reconstruction as shown in [6]. Hence, we need to rely on a more sophisticated reconstruction that has the ability to suppress noise in both the spatial and spectral domains and confidently identify and quantify the elemental distribution of each material. This is particularly important when looking to perform further spectral analyses, such as the use of K-edge subtraction (KES), where we segment elemental phases based on identification of their K-edges. For a detailed task-based reconstruction quality assessment based on KES analysis, we refer the reader to [6], where we compare more advanced spatio-spectral reconstruction methods for a biological sample.

## 6. Conclusions

Multichannel CT imaging opens up many new possibilities in material and life sciences. Multichannel CT is intrinsically ‘photon-hungry’ because detected photons are shared between multiple energy or time bins. Therefore, acquired tomographic datasets typically do not provide sufficient information for high quality reconstruction using traditional FBP-type algorithms. The absence of effective reconstruction methods and software capable of handling noisy and/or undersampled multichannel data hamper scientific applications of the technique.

The inverse problem framework provides methods to treat these challenging multichannel CT data through iterative reconstruction with suitable regularization which efficiently exploits prior knowledge and inter-channel correlation. CIL implements essential building blocks, which can explicitly support multichannel reconstruction and four—and higher—dimensional datasets. Here, we have demonstrated the potential of CIL for multichannel CT data with three representative case-studies. Starting with a simple colour denoising and inpainting problem, we illustrated the ability to incorporate various regularization techniques, such as classical TV, vectorial TV and TGV. We also outlined how a conventional formulation of iterative reconstruction through the optimization framework is mapped onto CIL objects. In the second case study, we exploited reconstructions on a dynamic sparse CT framework enforcing different prior information on spatiotemporal volume. We observed that spatio-temporal TV is able to remove noise and streak artefacts, but due to loss of contrast, important features are lost. Using a reference image from data with dense measurements, a structural prior (dTV) is shown to enhance the reconstructions when very low number of projections are acquired. To highlight the flexibility of CIL, we constructed both explicit and implicit PDHG to solve the corresponding reconstruction problems. Finally, in the last case study, we endeavoured to reconstruct an energy-resolved X-ray CT dataset with high energy resolution. We followed the same regularization strategy as in the second case study, i.e. a combination of edge preserving prior both in space and spectral directions, but this time we used a stochastic version of PDHG algorithm to speed-up large-scale CT reconstruction. Regularization aided better identification of K-edges in the energy-resolved X-ray CT dataset.

The ability to incorporate and balance various regularization terms in the reconstruction routine is a promising approach to treat noisy and undersampled multichannel CT data, especially when using different regularization strength for the spatial and energy domains. It is widely understood that one of the main challenges of iterative reconstruction is the high computational cost compared to traditional FBP-type methods. Although the focus of CIL is on modularity and on enabling the expression of complex optimization problems into working code, CIL wraps hardware accelerated libraries to perform costly forward- and back-projection steps and to calculate the proximal operators of regularization and fidelity terms, and there is a continuous effort to improve performance and resolve computational bottlenecks. In terms of supported imaging modalities, we can currently handle any tomographic modality which can be described by the Beer–Lambert law. We also provide interoperability with the Synergistic Image Reconstruction Framework (SIRF) [14] enabling positron emission tomography and magnetic resonance imaging reconstruction using CIL. We continue to enrich the library of available algorithms, regularizers, pre- and post-processing tools along with supported imaging models and available back-ends.

## Footnotes

1 $\bar{R}:=R\cup \{\infty \}$.

2 Image can be downloaded from https://github.com/TomographicImaging/CIL-Data.

## Data accessibility

Python scripts to reproduce the results for all the case studies are provided at [59] with CIL version 21.0 that is available through Anaconda; installation instructions are at https://www.ccpi.ac.uk/cil. In addition, CIL v21.0 and subsequent releases are archived at [5]. The gel phantom used in §4 can be found in [46]. The rock phantom used in §5 can be found in [58].
